# Local auxin competition explains fragmented differentiation patterns

**DOI:** 10.1038/s41467-020-16803-7

**Published:** 2020-06-11

**Authors:** Bernard Moret, Petra Marhava, Ana Cecilia Aliaga Fandino, Christian S. Hardtke, Kirsten H. W. ten Tusscher

**Affiliations:** 1 Department of Plant Molecular Biology, University of Lausanne, Biophore Building, CH-1015 Lausanne, Switzerland; 2 Theoretical Biology, Department of Biology, Utrecht University, 3584 CH Utrecht, The Netherlands

**Keywords:** Patterning, Auxin

## Abstract

Trajectories of cellular ontogeny are tightly controlled and often involve feedback-regulated molecular antagonism. For example, sieve element differentiation along developing protophloem cell files of *Arabidopsis* roots requires two antagonistic regulators of auxin efflux. Paradoxically, loss-of-function in either regulator triggers similar, seemingly stochastic differentiation failures of individual sieve element precursors. Here we show that these patterning defects are distinct and non-random. They can be explained by auxin-dependent bistability that emerges from competition for auxin between neighboring cells. This bistability depends on the presence of an auxin influx facilitator, and can be triggered by either flux enhancement or repression. Our results uncover a hitherto overlooked aspect of auxin uptake, and highlight the contributions of local auxin influx, efflux and biosynthesis to protophloem formation. Moreover, the combined experimental-modeling approach suggests that without auxin efflux homeostasis, auxin influx interferes with coordinated differentiation.

## Abstract (web summary)

Sieve element differentiation in *Arabidopsis* roots requires two antagonistic regulators of auxin efflux, BRX and PAX. Here the authors show that together they coordinate sieve element formation by preventing cell fate bistability emerging from AUX1-mediated competition for auxin between neighboring cells.

## Introduction

**Fig. 1:**
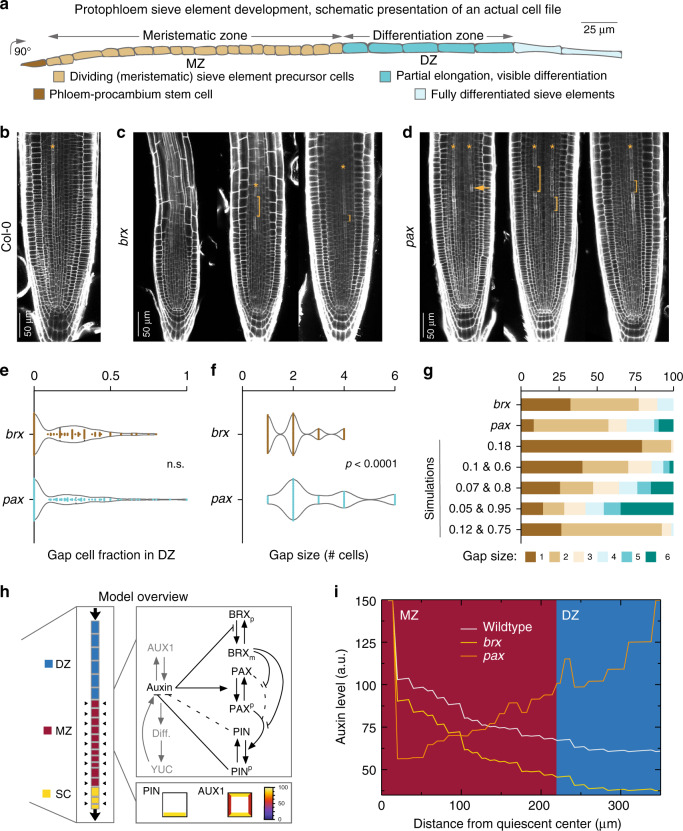
Antagonistic auxin efflux impairments trigger similar non-random protophloem differentiation failures. **a** Schematic overview of a developing protophloem sieve element (PPSE) cell file in the *Arabidopsis* root meristem. **b** Confocal microscopy image of a 7-day-old wild type (Col-0) root meristem, propidium iodide (PI) cell wall staining (protophloem cell file marked by an asterisk, as hereafter). **c**, **d** Phenotypic range of *brx* or *pax* mutant root meristems. Brackets point out protophloem “gaps”, i.e., PPSE precursors that fail to differentiate. Arrowheads highlight an isolated differentiated PPSE. **e**, **f** Quantification of gap cell frequency (**e**) and gap size (**f**) in indicated genotypes. **g** Comparison of experimentally observed simulated gap-size distributions. Simulation of y-axis values indicates differentiation failure probability of an individual cell, total or split, as a function of differentiation failure in the preceding cell. **h** Overview of the models developed in this study. Left: idealized PPSE strand (SC stem cells, MZ meristematic zone, DZ differentiation zone). Cellular PIN and AUX1 levels dictate auxin transport dynamics (shoot-derived auxin supplied to the differentiation zone via bulk phloem sap). The model incorporates cellular growth, division, early expansion, and differentiation dynamics, causing individual cells to move from the meristematic to the differentiation zone. Stem cells undergo slow, meristematic cells rapid divisions; differentiation zone cells undergo early phases of elongation. Right, top: individual model cells contain a regulatory network governing BRX membrane occupancy, PAX and PIN phosphorylation, and auxin efflux dynamics (black). This model network is incrementally augmented with auxin-dependent AUX1 expression, then differentiation, and finally differentiation-dependent YUCCA expression (gray). Right, bottom: individual model cells have a polar PIN pattern, and an apolar AUX1 pattern. **i** Steady-state auxin profiles in wild type, *brx*, and *pax* mutant settings in the initial PSSE model. Dark red indicates the meristematic zone, blue the differentiation zone. Discrete jumps in auxin levels reflect the transition between distinct cells. Within cells, more graded auxin changes occur. Plots display individual values (dots) and their density distribution. See Source Data for raw measurements and statistical test details.

Development of multicellular organisms entails tightly orchestrated cellular differentiation in response to temporal and spatial cues. Accordingly, trajectories of cellular ontogeny and their plasticity are under firm molecular-genetic control. Postembryonic plant development is highly plastic and modular in order to adapt to environmental conditions^1^. Nevertheless, once initiated, plant organs develop in stereotypic patterns, similar to animals^2^. Their sustained growth is driven by apical meristems, and requires phloem sap delivery from source organs^3,4^. Phloem sap contains sugars and other metabolites, as well as developmental signals, such as the phytohormone auxin^5^. It is transported through the phloem sieve tubes, which consist of interconnected sieve elements. In the sink tissues, for example in root apical meristems, the mature phloem sieve tubes connect to the early, so-called protophloem. Protophloem is continuously produced by the meristem’s stem cell niche, and is essential for meristem growth and maintenance^6^. In *Arabidopsis thaliana* root meristems, development of protophloem sieve elements (PPSEs) is laid out in a spatiotemporal gradient that comprises a meristematic zone where stem cell daughters divide, followed by a differentiation zone where elongating cells rearrange their cell walls and organelles, and eventually enucleate^6,7^ (Fig. 1a). The trajectory is overlaid by auxin accumulation around the stem cells, followed by gradual auxin decrease as cells divide and gradual auxin increase as they differentiate^8–10^. This auxin pattern emerges from polar auxin transport dynamics, with a key role for plasma-membrane-integral PIN-FORMED (PIN) auxin efflux carriers. PINs are rootward localized (i.e., at the plasma membrane that faces the root tip) in developing protophloem cells^11,12^, similar to most inner cell files^13,14^, and transport shoot-derived auxin delivered by bulk transport through mature phloem to the periphery of the meristem, as well as locally synthesized auxin, and auxin redirected by the root tip reflux loop^15,16^.

Controlled PIN activity is required for correct timing of PPSE differentiation^10,12^. This control is exerted by a “molecular rheostat” that connects two antagonistic regulators of auxin efflux, BREVIS RADIX (BRX) and PROTEIN KINASE ASSOCIATED WITH BRX (PAX)^12^. Both are polar plasma-membrane-associated proteins that co-localize with PINs. Whereas PAX stimulates PIN-mediated auxin efflux, BRX inhibits this activation. Because threshold auxin levels negatively regulate BRX plasma-membrane association and also stimulate PAX activity through phosphorylation^11,12,17^, a dynamic steady-state equilibrium ensues that fine-tunes PIN activity and thereby auxin flux through PPSE cell files. Yet, counterintuitively, both *brx* and *pax* loss-of-function mutants display discontinuous protophloem, which manifests in reduced root growth and other systemic effects^6,12,18^. This phenotype arises from seemingly stochastic failure of developing PPSEs to differentiate. Such cells stand out as morphological “gaps” that interrupt differentiation zone continuity (Fig. 1b–d). Here, we show that these patterning defects are distinct, non-random, and can be explained by a bistability in fate determination that emerges from competition for auxin between neighboring cells.

## Results and discussion

### Protophloem differentiation failures in *brx* and *pax* mutants show a non-random pattern

Upon phenotyping larger samples, we found that although the overall gap cell frequency was similar in *brx* and *pax* (Fig. 1e), larger (≥4-cell) gaps were significantly more abundant, and smaller (1-cell) gaps less abundant, in *pax* (Fig. 1f). Yet, in both genotypes, 2-cell gaps were most frequent. To investigate the nature of this pattern, we developed a simple 1D model that decided for each cell in a cell file independently whether it will become a gap cell or a differentiated cell. To simulate a random distribution, the chances of an individual cell to be assigned gap cell fate were set to the fraction of experimentally observed gap cells. This produced a gap cell distribution that was strongly skewed toward 1-cell gaps (Fig. 1g). These results suggest that loss of either auxin efflux inhibition or activation triggers distinct yet similar protophloem phenotypes that represent non-random disturbance of PPSE differentiation.

To determine which conditions could produce a preponderance of 2-cell gaps, we attributed a higher chance to attain gap fate if the preceding cell was a gap cell, while keeping the overall gap cell fraction constant. These conditions increased the frequency of 2-cell gaps, but also of larger-sized gaps beyond what was observed experimentally (Fig. 1g). Finally, predominantly 2-cell gaps without many larger gaps occurred when cells were assigned a higher chance to become a gap cell only if a single preceding cell was a gap cell (Fig. 1g). These combined results pointed to a potential interdependence of pairs of neighboring cells.

### Auxin flux acceleration or deceleration can trigger similar auxin-level reductions in the meristem

To investigate whether this could reflect auxin flux disturbance, we developed a mechanistic model for cellular auxin efflux regulation (Fig. 1h). In this model, ordinary differential equations describe the known auxin-dependent dynamics of BRX, PAX, and PINs (i.e., auxin-dependence of plasma membrane BRX levels and PAX activity, dependence of PIN activity on PAX activity, repression of PAX-mediated PIN stimulation by membrane-bound BRX, and auxin efflux resulting from PIN activation; see “Methods”). To investigate auxin efflux dependence on auxin levels, intracellular auxin levels were varied as a control parameter. *brx* and *pax* mutants were simulated by setting their gene product rates to zero. This model produced low steady-state PIN-mediated auxin export rates at low intracellular auxin levels, which increased with increasing auxin levels (Supplementary Fig. 1a). Virtual *brx* mutation did not simply lower the auxin level required to achieve the same efflux rate, but rather increased the minimum efflux rates at low auxin levels (Supplementary Fig. 1b), which can be understood from the dominant PAX baseline activity at lower auxin levels. In contrast, the absence of PAX resulted in constant low efflux rates, independent of cellular auxin level (Supplementary Fig. 1c).

We next implemented a spatial extension representing a developing PPSE strand that receives auxin from shootward (not explicitly modeled) mature phloem as well as lateral tissues (Fig. 1h). Assuming constant auxin influx as well as constant PIN levels, this model recreated auxin decrease away from the stem cell niche (Fig. 1i). In the virtual *brx* mutant, elevated auxin transport rates resulted in an overall reduction of auxin levels. In contrast, virtual *pax* mutation resulted in substantial auxin accumulation in shootward cells because of an auxin traffic jam arising in the absence of PIN phosphorylation, while more rootward cells displayed lower auxin levels. Thus, in both virtual mutants, a similar-sized integrated auxin reduction occurred across the spatial range of early meristematic cells (Fig. 1i). This could explain the paradoxically similar *brx* and *pax* phenotypes: whereas differentiation zone gaps are easy morphological readouts, incipient differentiation or specification might already fail in the meristematic zone^12^. However, the question how decreased auxin levels translate into the observed pattern of gap cells interspersed with normally differentiating cells remained open.

### Gap cell patterning depends on AUX1-mediated bistability and lateral inhibition

**Fig. 2:**
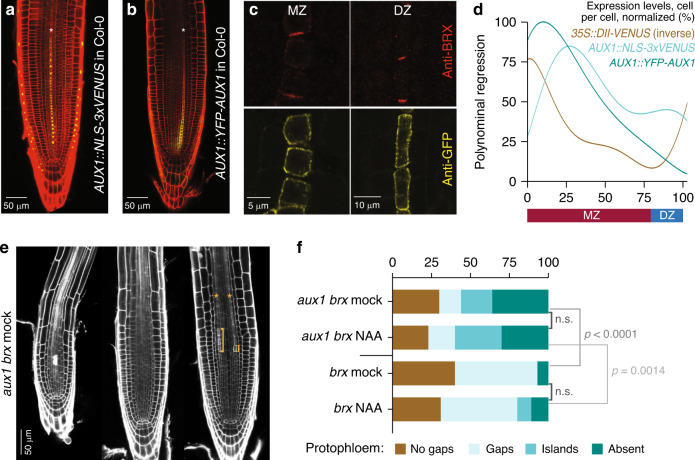
Intermittent gap cell patterning depends on the auxin influx facilitator AUX1. **a**, **b** Confocal imaging (PI staining, red) of *AUX1* transcriptional (**a**) and translational (**b**) reporter genes (yellow). **c** Anti-GFP immunostaining of YFP–AUX1 fusion protein (yellow) in developing PPSEs, with simultaneous anti-BRX staining (red) for PPSE identification. **d** Relative expression levels of *AUX1* reporters and the inverse auxin sensor DII-VENUS along developing PPSE cell files, fitted from experimental data (see Supplementary Fig. 3). **e** Phenotypic range of *aux1 brx* double-mutant root meristems, including isolated “islands” of differentiation (brackets). **f** Quantification of phenotype classes in *aux1 brx* double and *brx* single mutants, in mock conditions or upon treatment with 50 nM of the membrane-soluble synthetic auxin 1-naphthylacetic acid (NAA). Statistical significance of differences (chi-square test) are indicated. See Source Data for raw measurements and statistical test details.

**Fig. 3:**
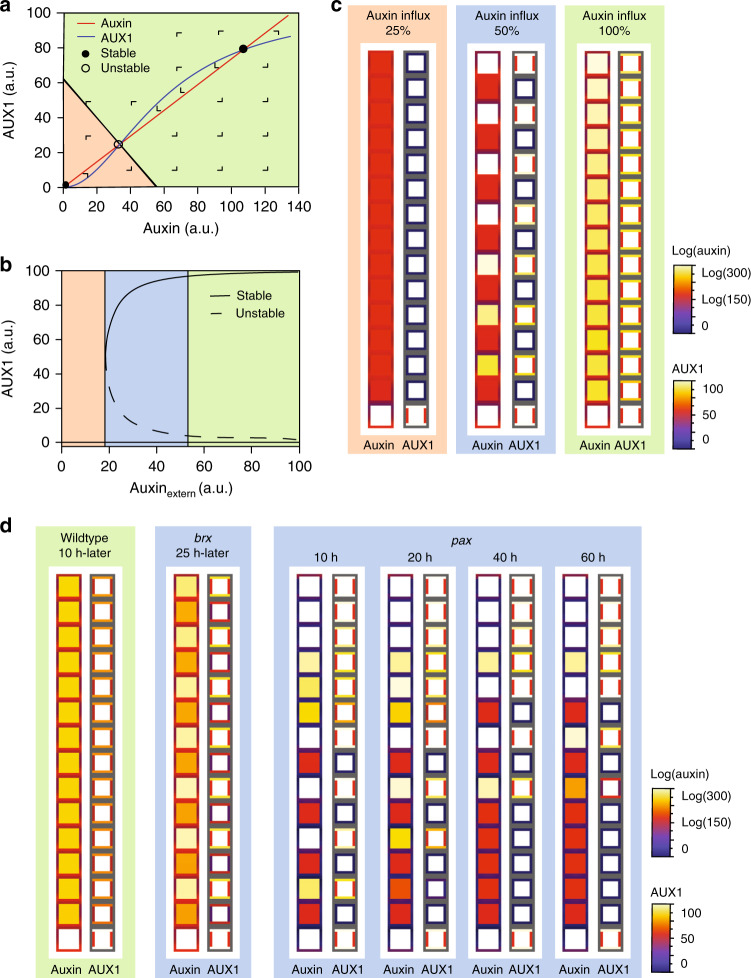
AUX1-dependent bistability. **a** Phase portrait of the auxin–AUX1 single-cell model for intermediate external auxin levels, showing auxin- and AUX1-null clines, the stable and unstable equilibrium intersection points, and the basins of attraction for low auxin-low AUX1 (LALA) (orange) and high auxin-high AUX1 (HAHA) (green) stable equilibria. **b** Bifurcation diagram of the auxin–AUX1 single-cell model using external auxin as the bifurcation parameter. Note the bistable parameter domain in which both the LALA and HAHA equilibria exist (blue). **c** Snapshots of steady-state auxin and AUX1 patterns in a 15-cell strand auxin–AUX1 model for varying levels of shoot-derived auxin influx. Background colors indicate correspondence with parameter regions shown in (**b**). **d** Snapshots for simulated *brx* mutants, *pax* mutants and wild type under constant shoot auxin influx (showing steady-state dynamics for *brx* and wild type, and patterning dynamics for *pax*).

The pattern suggested that individual developing PPSEs could be bistable, either attaining a stably differentiated or non-differentiated, gap cell state. Moreover, the intermittence of differentiated and non-differentiated cells suggested lateral inhibition-type patterning^19–21^, in which a state promotes itself while simultaneously repressing that same state in neighboring cells. An interesting candidate in this context is the auxin influx facilitator AUX1, whose auxin-dependent expression has been implicated in amplifying auxin patterning during lateral root initiation^22^ and root tropism^23^. AUX1 expression could cause lateral inhibition, because auxin import is further enhanced through AUX1 induction, thereby generating the positive feedback that is essential for bistability, while auxin uptake of a particular cell depletes directly neighboring cells of that same auxin. Besides the columella-root cap, where it is required for root gravitropism, AUX1 is specifically expressed in developing protophloem^24^, all around PPSEs (Fig. 2a–c, Supplementary Fig. 2a), essentially apolar but with possibly slightly higher abundance along the apical–basal axis (Supplementary Fig. 2b, c). *aux1* loss-of-function mutants do not display discernible root phenotypes apart from agravitropism^25^. However, *aux1* mutation exacerbates the *brx* phenotype^12^. In ca. one-third of *aux1 brx* double mutants, distinguishable protophloem was missing, while otherwise an “inverse gap phenotype” of isolated differentiated cells among mostly undifferentiated cells occurred frequently (Fig. 2e, f). To investigate whether AUX1 could affect PPSE bistability, we developed a second differential equation-based single-cell model that focused on the interplay between intracellular auxin and AUX1. For simplicity, we assumed constant, PIN-mediated auxin efflux, and incorporated dependence of AUX1 levels on intracellular auxin (Fig. 2d, Supplementary Fig. 3a–d), as well as AUX1-dependent auxin uptake. To investigate potential bistability of intracellular auxin and AUX1 levels, we varied external auxin levels as a control parameter. When the experimentally observed nonlinear positive dependence of AUX1 on auxin levels was implemented, this model created two alternative stable states separated by an auxin threshold. Cells that started out with below-threshold auxin levels converged on a low auxin-low AUX1 (LALA) state, and cells that started out with above-threshold auxin levels converged on a high auxin-high AUX1 (HAHA) state (Fig. 3a). Bistability only arose at intermediate external auxin levels (Fig. 3b), with lower or higher auxin resulting in exclusive accessibility of the LALA or HAHA state, respectively.

**Fig. 4:**
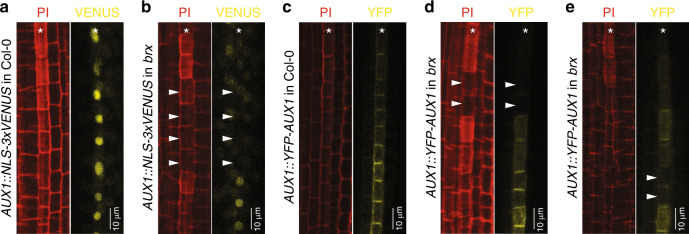
AUX1 expression in *brx* mutants. **a**–**e** Confocal microscopy images of developing PPSE cell files (asterisks); left panels: propidium iodide cell wall staining (red); right panels: reporter fluorescence (yellow). **a**, **b** Transcriptional reporter of *AUX1* gene expression in developing PPSEs of Col-0 wild type (**a**) or *brx* (**b**). **c**–**e** Expression of YFP–AUX1 fusion protein in wild type (**c**), the *brx* differentiation zone (**d**), and the *brx* early meristematic zone (**e**). Gap cells in *brx* are marked by arrowheads in **b** and **d**, cells with low AUX1 are marked by arrowheads in **e**.

In agreement, three alternatives emerged in a simple spatial model extension to a cell file (Fig. 3c). At low auxin availability, all cells converged to a LALA state. At higher external auxin availability, all cells converged to a HAHA state. However, intermediate levels of auxin availability resulted in an alternating pattern of HAHA and LALA cells, because AUX1-mediated auxin influx mostly impaired auxin uptake capacity of directly neighboring cells. Next, we kept auxin influx constant and implemented BRX- and PAX-regulated PIN activity. In this model, simulated *brx* and *pax* mutations resulted in alternating HAHA–LALA phenotypes (Fig. 3d), consistent with the limited auxin-level decrease they induced in the earlier PPSE-strand model. Still, whereas virtual *brx* mutants showed highly regular and temporally constant HAHA–LALA patterning, virtual *pax* mutants displayed a temporally variable, alternating pattern rootward, with a stretch of HAHA cells shootward. In summary, the modeling suggested that local auxin reductions in both *brx* and *pax* protophloem cause AUX1-dependent competition for auxin, and generate intermittent HAHA–LALA patterning. These results matched experimental observations. For example, both *AUX1* transcription and AUX1 protein levels were typically reduced in *brx* gap cells (Fig. 4a–d). Moreover, local AUX1 reductions were also frequently observed in the *brx* meristematic zone (Fig. 4e). These findings were also consistent with reported higher fluctuation of cellular auxin levels along developing *brx* protophloem^12^, and lower auxin content in *brx* gap cells as determined by the DII-VENUS auxin sensor (Supplementary Fig. 4a–d). Finally, to confirm the causal role of AUX1 rather than mere auxin availability in creating the bistability of developing PPSEs, we treated *aux1 brx* double mutants with a membrane-diffusible synthetic auxin that does not require active import. Consistent with our model predictions, this treatment could not revert *aux1 brx* double mutants to the typical *brx* single-mutant gap pattern phenotype (Fig. 2f).

### Early meristematic fate specification drives gap–non-gap distribution

**Fig. 5:**
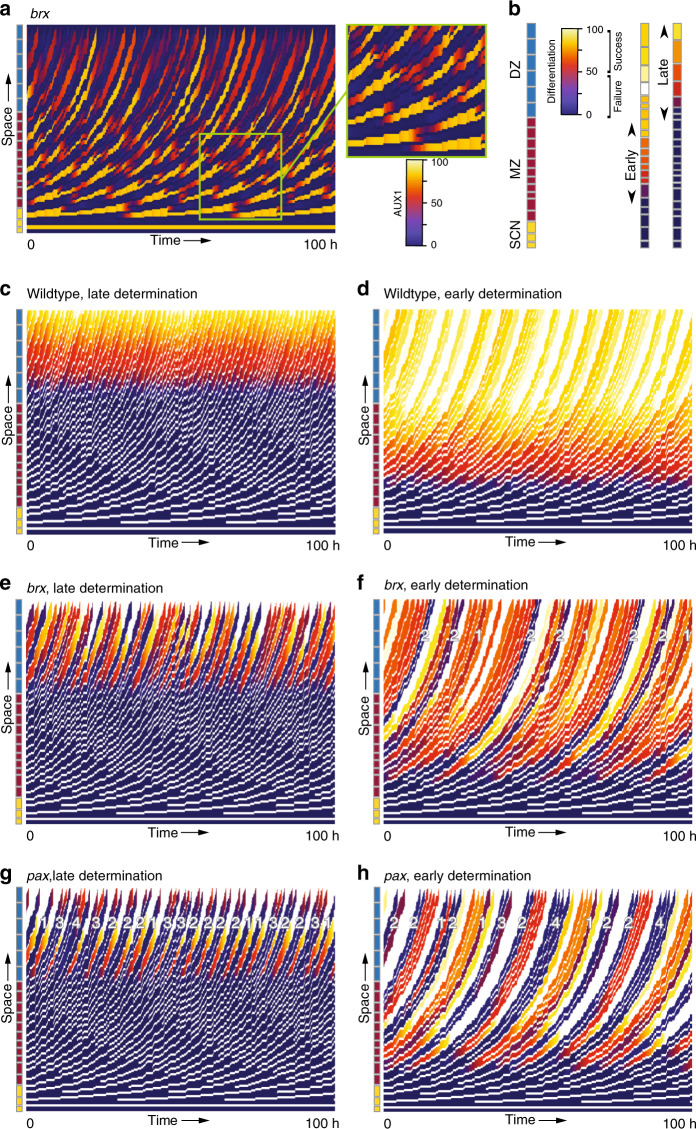
Early fate determination explains differentiation patterns. **a** Kymograph of cellular AUX1 expression levels for a *brx* mutant in the PPSE-strand model. The inset shows details of AUX1 dynamics in the meristematic zone, highlighting the transient presence of single, double-sized cells just prior to division, and slightly later divided cell pairs with differential AUX1 expression. **b** Snapshots of differentiation dynamics for wild type in the case of early and late fate determination, respectively. Arrows indicate the PPSE zone in which fate is determined for the respective differentiation scenarios. **c**–**g** Differentiation kymographs for wild type, *brx*, and *pax* mutants in the two scenarios. Numbers in **e**–**h** indicate gap sizes. For *brx*, only 1-cell gaps (not marked) are observed in the late scenario.

While models incorporating AUX1 generated intermittent gaps, these regular, 1-cell gaps did not match observations. However, because of the inherent tendency for alternation, this pattern was substantially perturbed once we moved to the PPSE-strand model that incorporated divisions. Upon cell division, single LALA (or HAHA) cells were transiently transformed into a LALA (or HAHA) pair, followed by dynamic readjustment of auxin–AUX1 patterning (Fig. 5a). Based on this observation of transient cell pairs, we hypothesized that the timing of fate determination onset may be important for gap cell patterning. We therefore refined the PPSE-strand model by including an abstract description of auxin-dependent differentiation, where cells that remain below a certain threshold level of the simulated differentiation factor become gap cells, whereas cells that pass this threshold will become PPSEs. The latter is consistent with the observation that PPSE differentiation can no longer be suppressed, once a cell has committed to this fate^6,26^. In the first scenario, we assumed that fate determination occurs once cells are distant enough from the stem cell niche (early scenario), and is fixed when cells enter the differentiation zone. In an alternative scenario, we assumed that fate is only determined once cells exit the meristematic zone (late scenario). While approximately similar final differentiation levels were achieved in both wild type scenarios (Fig. 5b–d), clear differences arose for the virtual *brx* mutant: whereas the late scenario produced an alternating 1-cell gap pattern (Fig. 5e), the early scenario regularly produced the experimentally observed 2- as well as 1-cell gaps (Fig. 5f). Moreover, consistent with experimental observations, gaps were interspersed with larger numbers of differentiated cells. This asymmetry resulted from the different dynamics of auxin and AUX1 versus differentiation. Whereas auxin and AUX1 levels can both increase and decrease, differentiation merely increases, albeit at considerably slower rates for lower auxin levels. As a consequence, daughter cells of early meristematic HAHA cells start with a differentiation head start, and although division-induced LALA–HAHA repatterning (Fig. 5a) may cause auxin and AUX1 levels to decrease at later stages, they mostly differentiate (Fig. 5f). In contrast, daughter cells of early meristematic LALA cells start out with a differentiation delay, and only those that gain higher auxin early enough in the meristem due to division-induced LALA–HAHA repatterning differentiate, while others become gap cells (Fig. 5f). Thus, early meristematic LALA cells produced both gap cell pairs and differentiated daughter cells, whereas early meristematic HAHA cells produced mostly differentiated cells (Fig. 5f).

Unlike the virtual *brx* mutant, the virtual *pax* mutant produced a large number of 1-, 2-, and 3-cell gaps in the late scenario (Fig. 5g). In the early scenario, virtual *pax* regularly produced 2- as well as 4-cell gaps interspersed with larger non-gap regions (Fig. 5h), matching experimental observations. These dynamics were more easily observed when a positive feedback of differentiation on itself was incorporated (Supplementary Fig. 5a–c). Note that division frequency or asynchrony were not tuned to more precisely match our models, since experimental data to realistically constrain these parameters are currently unavailable. Yet, our models provide proof-of-principle that early fate determination is necessary for a 2-cell gap pattern interspersed with longer stretches of differentiated cells, matching the experimentally observed early loss of distinct PPSE markers^12^.

### Auxin flux homeostasis buffers auxin level fluctuations

To gauge the role of auxin levels and sources, we next investigated local biosynthesis. Interestingly, we found that among potentially protophloem-expressed *YUCCA* genes, which encode the rate-limiting enzymes in auxin biosynthesis^27,28^, only *YUC5* was expressed in the protophloem, specifically in the differentiation zone (Supplementary Fig. 3e–f; Supplementary Fig. 6a). However, *yuc5* loss-of-function mutants displayed at best somewhat delayed PPSE differentiation (Supplementary Fig. 6b), but no gap or root phenotype. *yuc5* mutation also did not enhance *brx* defects (Supplementary Fig. 6c), consistent with strongly reduced *YUC5* expression in *brx* protophloem (Supplementary Fig. 6d). Although we could not determine whether auxin levels in protophloem are indeed reduced in *yuc5* mutants, these effects were reproduced by our model, and could be ascribed to an overall lower auxin level (Supplementary Fig. 7a–d). This indicates that mere reduction of auxin level through either reduced production (Supplementary Fig. 7b) or uptake (Supplementary Fig. 7e, f) is insufficient to generate gap cells. Supporting this notion, simulations of auxin reduction in wild type merely delayed differentiation (Supplementary Fig. 7g). A functional auxin efflux homeostasis caused auxin efflux to increase with higher and decrease with lower intracellular auxin levels, and thereby counteracted AUX1-mediated competition for auxin and hence prevented bistability. On a similar note, the failure to rescue *brx* mutants by application of a synthetic membrane-diffusible auxin indicated that mere auxin addition is insufficient to prevent gap cell formation (Fig. 2f). Again, these results were reproduced by our model (Supplementary Fig. 7h), and can be explained by the cells’ diminished capacity to hold on to auxin in the absence of BRX, and hence its incapacity to prevent AUX1-mediated bistability. Collectively, the results thus indicate that gap cells emerge from a combination of auxin-level reduction and absent flux homeostasis, which triggers AUX1-induced competition for auxin and causes bistable cell fate acquisition. This also means that the BRX–PAX auxin flux rheostat protects the protophloem from differentiation failure when auxin levels fluctuate, by increasing efflux from high auxin-content cells and decreasing efflux from low auxin-content cells.

In summary, our combined experimental-modeling approach explains the paradoxical observation that both genetic flux enhancement and repression trigger a similar protophloem phenotype. The data suggest that once auxin availability and flux homeostasis are severely disrupted, cells that lose out in competition for auxin spiral into a low auxin uptake capacity state that prevents their timely differentiation. In this scenario of auxin-dependent bistability, facilitator-driven auxin uptake gains in importance, and explains the non-random differentiation failure pattern observed in the mutants. Yet, our findings also corroborate that as long as auxin efflux homeostasis is functional, impaired auxin influx or local biosynthesis have little effect on differentiation capacity. It appears possible however that auxin influx facilitation into developing protophloem gains importance in more challenging conditions than unobstructed root growth in tissue culture, for example upon extreme root tip bending to avoid obstacles in the soil. Here, it might protect developing protophloem from the dynamic, adaptive auxin flux adjustments throughout the root meristem by maintaining its auxin sourcing and thereby proper differentiation of an essential tissue.

## Methods

### Plant material and growth conditions

The *A. thaliana* wild type line used in this study was Col-0, which was also the genetic background for the mutants and transgenic lines. For plant tissue culture, seeds were surface-sterilized, stratified for 2 days in the dark at 4 °C, and germinated in vertically placed Petri dishes on 0.7% agar and 0.5× Murashige and Skoog (½ MS) medium (Duchefa) with 0.3% sucrose at 22 °C under continuous light. The following transgenic and mutant lines have been described before: *aux1–7* and *AUX1::YFP–AUX1*^29^*, brx*, *aux1–7 brx-2*, and *pax*^12^.

### Generation of constructs, transgenic lines, and *yuc5* mutants

Transgenes for plant transformation were created in suitable binary vectors and produced through standard molecular biology procedures and/or NEBuilder HiFi DNA Assembly Reaction Protocol. For the cloning of the *pX::NLS-3xVENUS* constructs, the following primer combinations were used to insert the amplified promoters into a version of the pCAMBIA1305.1 binary vector containing a *NLS-3xVENUS* reporter downstream of a multicloning site. The restriction sites that were used were KpnI or Eco53kI for the forward primers and SbfI for the reverse primers (Supplementary Table 1). The *yuc5* mutant lines were generated using the CRISPR/Cas9 technology and targeting the *YUC5* reference sequence (AT5G43890). Guides were designed with the help of the CRISPR-P website (http://crispr.hzau.edu.cn/cgi-bin/CRISPR2/CRISPR) (Supplementary Table 2).

### Plant transformation

The binary constructs were introduced into *Agrobacterium tumefaciens* strain GV3101 pMP90 and transformed into *A. thaliana* using the standard floral dip method. At least three independent transgenic lines were used for each construct to perform experiments and verify reproducibility.

### Protein immunolocalization

Immunostaining was performed on 5-day-old seedlings as previously described^11^. Samples were imaged by confocal laser-scanning microscopy. YFP–AUX1 fusion protein was detected with rabbit polyclonal anti-GFP antibody (Abcam, Cat# ab290; dilution 1:500), and endogenous AUX1 was detected with goat polyclonal anti-AUX1 antibody (Agrisera, Cat# AS16 3957; dilution 1:600). Alexafluor anti-rabbit/goat 546 donkey secondary antibodies (Molecular Probes; dilution 1:500) were used for primary antibody detection.

### Microscopy

To visualize reporter genes and staining signals, fluorescence for VENUS (excitation 515 nm, emission 528 nm), YFP (excitation 512 nm, emission 529 nm), and propidium iodide (excitation 536 nm, emission 617 nm) was detected in seedlings examined under a Zeiss LSM 700 inverted confocal scanning microscope. Seven days after germination, seedlings were used for quantifications. For presentation, composite images had to be assembled in various instances. Sequential scanning was used to avoid any interference between fluorescence channels of simultaneously detected probes. For image analyses, ImageJ (NIH; https://rsb.info.nih.gov/ij) and Zeiss Zen (black edition) were used.

### Modeling

To investigate whether gap cells follow a random distribution or not, we developed a gap-frequency model, consisting of a one-dimensional strand of cells, the length of a typical protophloem cell file, in which individual cells could be assigned normal or gap cell fate, such that the final gap cell frequencies fit experimental observations in *brx* and *pax* mutants. We varied whether the chances of individual cells to be assigned gap cell fate depend on the fate of preceding cells or not. To investigate how the interplay between BRX, PAX, and PIN impacts auxin flux and patterning, we developed a single-cell ordinary differential equation-based model (parameter settings described in Supplementary Table 3), and subsequently extended this to a one-dimensional protophloem tissue-strand model incorporating cell growth, division, expansion, and differentiation. To investigate auxin-dependent AUX1 expression-mediated bistability, we developed a second ordinary equation-based model (parameter settings described in Supplementary Table 4), which subsequently was incorporated first in a simplified, non-growing, non-zonated tissue-strand model, and then in the protophloem tissue-strand model. Parameter settings for the protophloem tissue-strand model and auxin dynamics (Supplementary Fig. 8) are described in Supplementary Table 5. For a full description of the models including equations see Supplementary Methods.

### Reporting summary

Further information on research design is available in the Nature Research Reporting Summary linked to this article.

## Supplementary information

Supplementary Information

Reporting Summary

## Data Availability

All data in this study are available in the main text or the Supplementary materials. This file also contains the statistical test details. Source data are provided with this paper.

## Source data

Source Data

## Code availability

The source codes for the different models described here are available on http://bioinformatics.bio.uu.nl/khwjtuss/ProtophloemModel.

## References

[1] Sultan SE (2000). Phenotypic plasticity for plant development, function and life history. Trends Plant Sci..

[2] Halle F (1986). Modular Growth in Seed. Plants Philos. Trans. R. Soc. B.

[3] Holbrook NM, Knoblauch M (2018). Editorial overview: physiology and metabolism: phloem: a supracellular highway for the transport of sugars, signals, and pathogens. Curr. Opin. Plant Biol..

[4] Milne RJ, Grof CP, Patrick JW (2018). Mechanisms of phloem unloading: shaped by cellular pathways, their conductances and sink function. Curr. Opin. Plant Biol..

[5] Teale WD, Paponov IA, Palme K (2006). Auxin in action: signalling, transport and the control of plant growth and development. Nat. Rev. Mol. Cell Biol..

[6] Rodriguez-Villalon A (2014). Molecular genetic framework for protophloem formation. Proc. Natl Acad. Sci. USA.

[7] Furuta KM (2014). Plant development. Arabidopsis NAC45/86 direct sieve element morphogenesis culminating in enucleation. Science.

[8] Brunoud G (2012). A novel sensor to map auxin response and distribution at high spatio-temporal resolution. Nature.

[9] Sabatini S (1999). An auxin-dependent distal organizer of pattern and polarity in the Arabidopsis root. Cell.

[10] Santuari L (2011). Positional information by differential endocytosis splits auxin response to drive Arabidopsis root meristem growth. Curr. Biol..

[11] Marhava P (2020). Plasma membrane domain patterning and self-reinforcing polarity in arabidopsis. Dev. Cell.

[12] Marhava P (2018). A molecular rheostat adjusts auxin flux to promote root protophloem differentiation. Nature.

[13] Adamowski M, Friml J (2015). PIN-dependent auxin transport: action, regulation, and evolution. Plant Cell.

[14] Petrasek J, Friml J (2009). Auxin transport routes in plant development. Development.

[15] Blilou I (2005). The PIN auxin efflux facilitator network controls growth and patterning in Arabidopsis roots. Nature.

[16] Grieneisen VA, Xu J, Maree AF, Hogeweg P, Scheres B (2007). Auxin transport is sufficient to generate a maximum and gradient guiding root growth. Nature.

[17] Scacchi E (2009). Dynamic, auxin-responsive plasma membrane-to-nucleus movement of Arabidopsis BRX. Development.

[18] Anne P, Hardtke CS (2018). Phloem function and development-biophysics meets genetics. Curr. Opin. Plant Biol..

[19] Greenwald I, Rubin GM (1992). Making a difference: the role of cell-cell interactions in establishing separate identities for equivalent cells. Cell.

[20] Oster GF (1988). Lateral inhibition models of developmental processes. Math. Biosci..

[21] Raible DW, Eisen JS (1995). Lateral specification of cell fate during vertebrate development. Curr. Opin. Genet. Dev..

[22] Laskowski M (2008). Root system architecture from coupling cell shape to auxin transport. PLoS Biol..

[23] van den Berg T, Korver RA, Testerink C, Ten Tusscher KH (2016). Modeling halotropism: a key role for root tip architecture and reflux loop remodeling in redistributing auxin. Development.

[24] Swarup R (2001). Localization of the auxin permease AUX1 suggests two functionally distinct hormone transport pathways operate in the Arabidopsis root apex. Genes Dev..

[25] Bennett MJ (1996). Arabidopsis AUX1 gene: a permease-like regulator of root gravitropism. Science.

[26] Hazak O (2017). Perception of root-active CLE peptides requires CORYNE function in the phloem vasculature. EMBO Rep..

[27] Stepanova AN (2011). The Arabidopsis YUCCA1 flavin monooxygenase functions in the indole-3-pyruvic acid branch of auxin biosynthesis. Plant Cell.

[28] Zhao Y (2001). A role for flavin monooxygenase-like enzymes in auxin biosynthesis. Science.

[29] Swarup R (2004). Structure-function analysis of the presumptive Arabidopsis auxin permease AUX1. Plant Cell.

