# Supplementary material for: Local auxin competition explains fragmented differentiation patterns
Source: Nat Commun. 2020 Jun 11;11:2965. doi: 10.1038/s41467-020-16803-7 (PMC7289883; doi:10.1038/s41467-020-16803-7)
Supplement: Supplementary file 1 — Supplementary Information [file 41467_2020_16803_MOESM1_ESM.pdf]

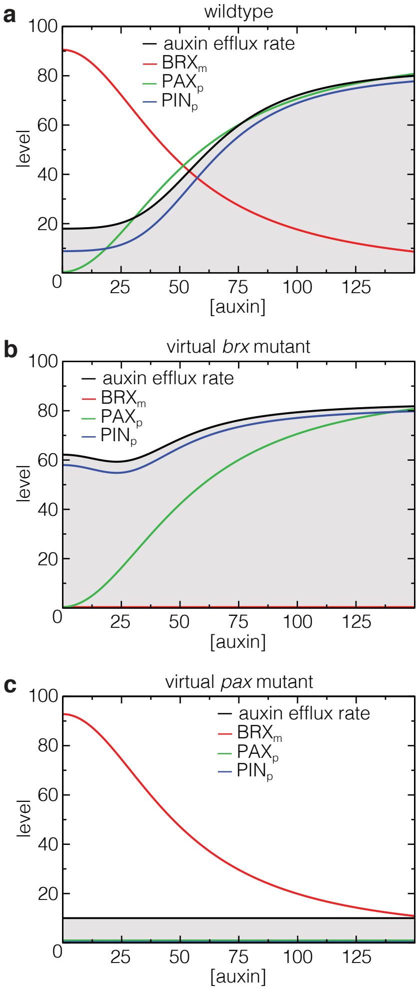

Supplementary Fig. 1. Single cell model of the BRX-PAX auxin efflux rheostat. Steady-state membrane-associated BRX (BRX_m), phosphorylated PAX (PAX_p) and PIN (PIN_p) levels, and resulting PIN-mediated auxin efflux, as a function of internal auxin level for simulated **a**, wildtype, **b**, *brx* mutant, and **c**, *pax* mutant.

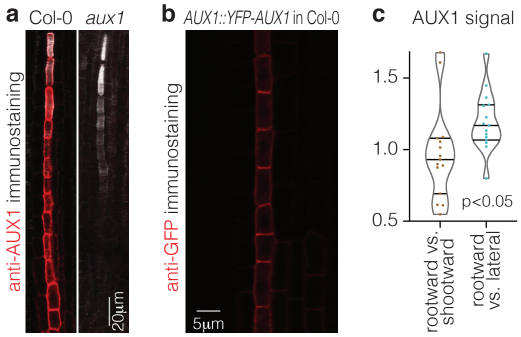

Supplementary Fig. 2. Apolar AUX1 localization in PPSEs. a, Detection of endogenous AUX1 in developing PPSEs of Col-0 wildtype with anti-AUX1 immunostaining, overlaid with calcofluour white cell staining (grey). A staining of the *aux1-21* null mutant is shown as a negative control. **b,** YFP-AUX1 detection by anti-GFP immunostaining in developing PPSEs. Note seemingly higher AUX1 levels shootward and rootward due to adjacency of the membranes. **c,** Quantification AUX1 signal ratios between shoot-/rootward and lateral membranes, obtained exclusively from detached PPSEs. Plots display individual values (dots) and their density distribution. See Source Data for raw measurements and statistical test details.

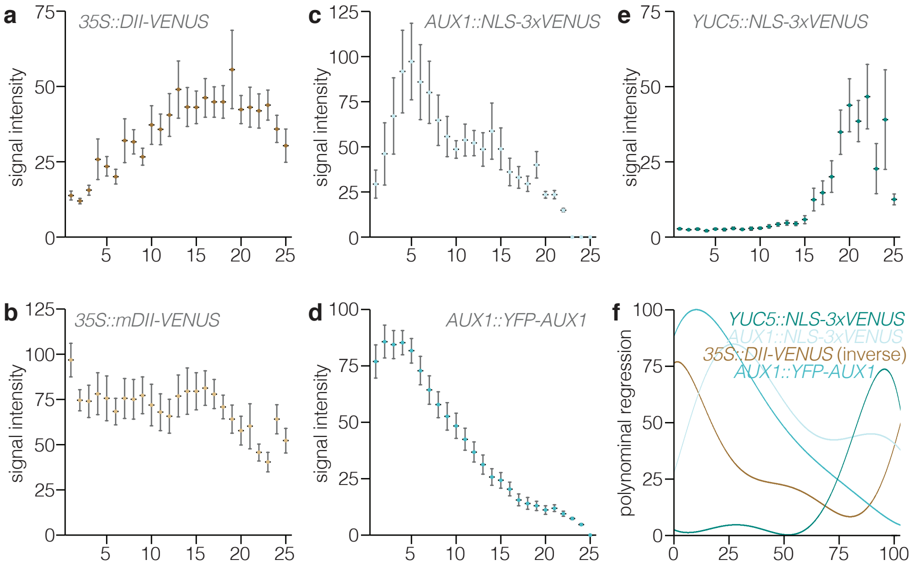

Supplementary Fig. 3. Expression profiles in PPSEs. a-e, Quantification of reporter gene expression levels along developing PPSE cell files, determined cell-by-cell in pertinent regions of interest (nucleus or plasma membrane). DII-VENUS is an inverse reporter of cellular auxin levels, mDII-VENUS its inert control. **f,** Fitted polynominal regression curves for the expression patterns, normalized along the cell files for the spread between highest (100%) and lowest (0%) raw expression values. See Source Data for raw measurements.

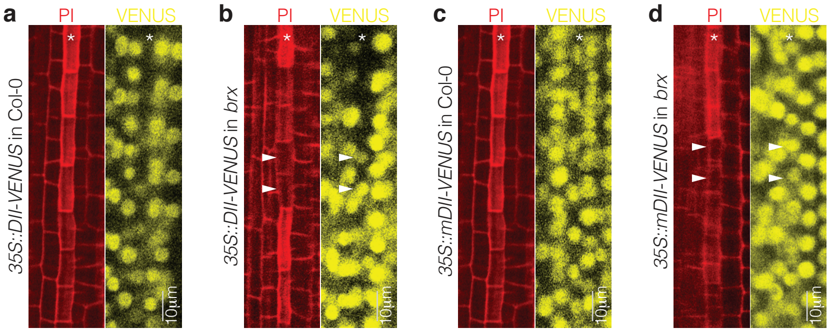

Supplementary Fig. 4. DII-VENUS expression in developing PPSEs. a-d, Confocal microscopy images of developing PPSE cell files (asterisks); left panels: propidium iodide cell wall staining (red); right panels: reporter fluorescence (yellow). **a-b,** Expression of the inverse auxin sensor DII-VENUS in developing PPSEs of Col-0 wildtype (a) or *brx* (b). **c-d,** Corresponding expression of the auxin-insensitive negative control reporter protein mDII-VENUS. Gap cells in *brx* are marked by arrowheads in b and d.

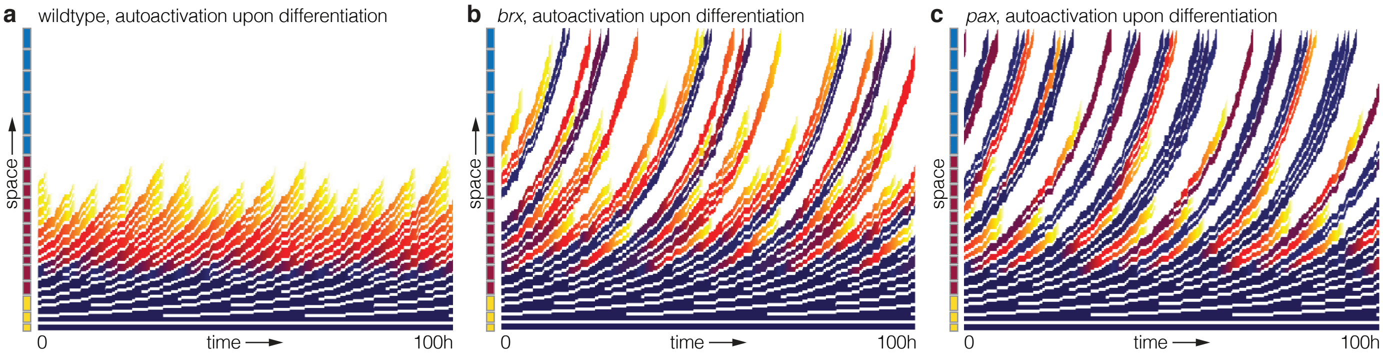

Supplementary Fig. 5. Early specification explains differentiation patterns. a-c, Differentiation kymographs for wildtype, *brx* and *pax* mutants in the early scenario (see Figure 5), with additional implementation of a positive feedback of differentiation on itself.

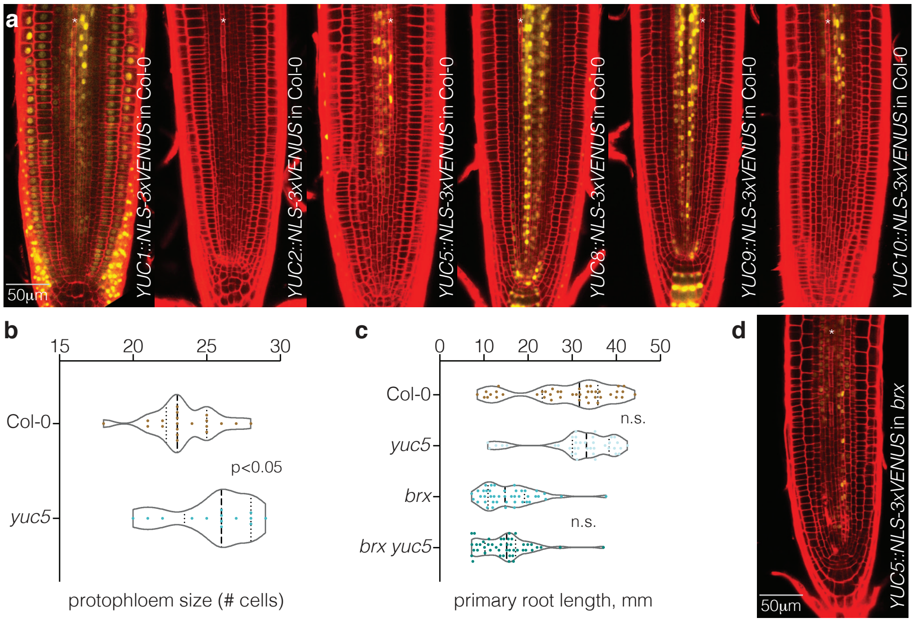

Supplementary Fig. 6. Local auxin biosynthesis in developing PPSEs. a, Confocal imaging (PI staining, red) of transcriptional reporters (yellow) for different YUCCA genes in Col-0 wildtype. **b,** Protophloem cell file length (counted from the first cell adjacent to the QC up to the last cell of the differentiation zone) in Col-0 and a *yuc5* knock-out mutant. **c,** Root length for 7-day-old seedlings of indicated genotypes. **d,** Confocal imaging of the transcriptional *YUC5* reporter in *brx* mutant background. Plots display individual values (dots) and their density distribution. See Source Data for raw measurements and statistical test details.

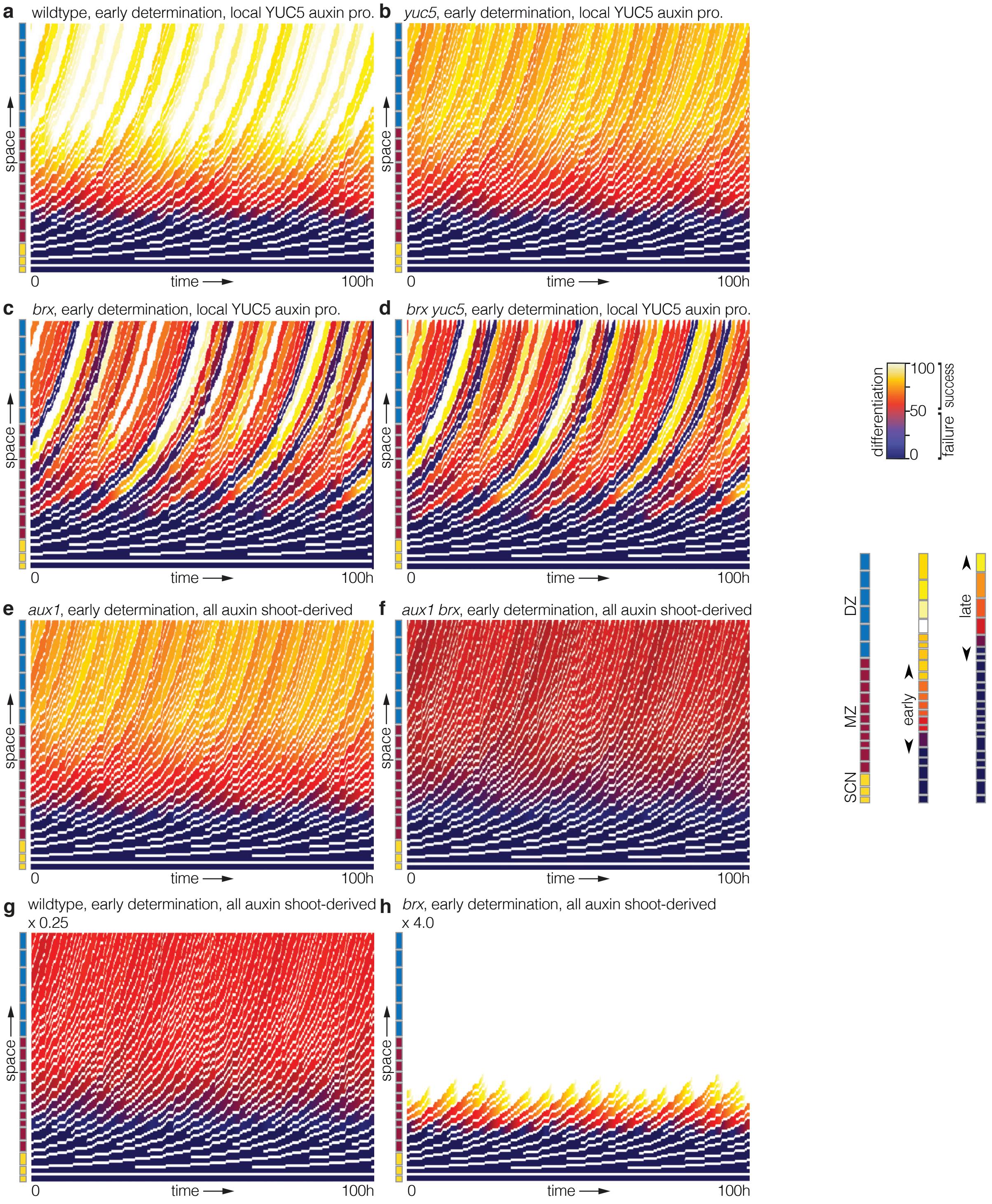

Supplementary Fig. 7. PPSE differentiation as a function of local auxin biosynthesis. a-h, Differentiation kymographs for indicated simulated genotypes, in the early scenario.

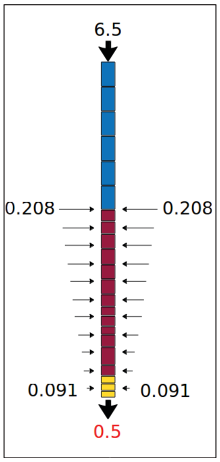

Supplementary Fig. 8. Overview of auxin influx and efflux into the simulated protophloem strand. Top influx, bottom efflux (fat arrows), emulated reflux loop influx (normal arrows). Influx/efflux rates inducated with black/red numbers.

	Forward primer	Reverse primer
pAUX1 (KpnI/SbfI)	5'- CTC GGT ACC CAA GAG ATT TTG AAG ACT CTT C -3'	5'- CAG CC TGC AGG TTT TTT AGC TTC TAG ATC TGA GA -3'
pYUC1 (Eco53KI/SbfI)	5'- CTC GAG CTC AAG CTT AAC AAA CTG CAA GGA AGT G -3'	5'- CAG CC TGC AGG TCT TGA TGG ATG ATG GAA AAT GTT TTA AA -3'
pYUC2 (Eco53KI/SbfI)	5'- CTC GAG CTC TTA GCA TAA TCA AAT TTT AGT TAC TAG AC -3'	5'- CAG CC TGC AGG GAA AGA GAG AAA GAG AAG AAA AAA GA -3'
pYUC5 (Eco53KI/SbfI)	5'- CTC GAG CTC CTA CAA CTA CAA AGG GAG CTT TC -3'	5'- CAG CC TGC AGG CTT TAG GGG TGA GTT TGA TCG A -3'
pYUC8 (Eco53KI/SbfI)	5'- CTC GAG CTC GCA TAT ATA AGG TTC TAC CAC GA -3'	5'- CAG CC TGC AGG TCT TTT TTT ATA AGT TTC TTT AAT AAG TAT TG -3'
pYUC9 (Eco53KI/SbfI)	5'- CTC GAG CTC AGA CGA TCA CTG AAC CTA ACC -3'	5'- CAG CC TGC AGG TTT CTT GAG TGA GTT TTT GAA TGA AAG -3'
pYUC10 (Eco53KI/SbfI)	5'- CTC GAG CTC GTA AAG TGA CTA ATT TTC CAA TTA AGT T -3'	5'- CAG CC TGC AGG TTC TTG TGT TTA GTT TGA TAG ATT CTC -3'

Supplementary Table 1: List of the primers used for promoter amplification prior to integration into *NLS-3xVENUS* pCAMBIA1205.1 construct templates.

ID	Score	Sequence		%GC
guide2	0.849	5'-ATA TTC CAT CTC CGA CCC CGA GG-3'	CDS	55%
guide5	0.8146	5'-AGT CAC GTG GAG CTA GTA GAC GG-3	CDS	50%
guide9	0.7393	5'-CAC GGC AGC CTG CCT CCG CGA GG-3	CDS	80%

Supplementary Table 2: List of the RNA guides used for the generation of the *yuc5* mutant lines.

Parameter	Meaning	Value	Units
BRX_{tot}	Total cellular amount of BRX protein	100	[ ]
brx_{exo}	Maximum rate of BRX exocytosis	0.1	s^{-1}
brx_{endo}	Rate of BRX endocytosis	0.01	s^{-1}
K_{brx}	Auxin leval at which exocytosis rate is half maximal	15	[ ]
PAX_{tot}	Total cellular amount of PAX protein	100	[ ]
pax_{act}	Maximum rate of PAX phosphorylation	0.1	s^{-1}
pax_{deact}	Rate of PAX dephosphorylation	0.01	s^{-1}
K_{pax}	Auxin level at which phosphorylation rate is half maximal	180	[ ]
$PIN1_{tot}$	Total cellular amount of PIN1 protein	100	[ ]
$pin1_{act}$	Maximum rate of PIN1 phosphorylation	0.1	s^{-1}
$pin1_{deact}$	Rate of PIN1 dephosphorylation	0.001	s^{-1}
$K_{PIN1,pax}$	(weighted) PAX level at which PIN1 phosphorylation rate is half maximal	250	[ ]
$K_{Pin1,brx}$	BRX level at which PIN1 phosphorylation rate is half maximal	25	[ ]
enh_{paxp}	Relative effectiveness of PAXp versus PAX	2.5	dimensionless
enh_{pinp}	Relative effectiveness of PINp versus PIN	10	dimensionless

Supplementary Table 3: Default parameter values for the single cell BRX PAX PIN model.

Parameter	Meaning	Value	Units
p_{aux1}	Maximum rate of AUX1 production	0.04	$[\ \ ]s^{-1}$
d_{aux1}	Rate of AUX1 degradation	0.0004	s^{-1}
K_{aux1}	Auxin level at which AUX1 production is half maximal	60	$[\ \ ]$
i_{aux1}	Rate of active AUX1 dependent auxin influx	0.20	$[AUX1]^{-1}s^{-1}$
$PIN1$	Total cellular amount of PIN1 protein	100	$[\ \ ]$
e_{pin1}	Rate of active PIN1 dependent efflux	0.04	$[PIN1]^{-1}s^{-1}$
p_{auxin}	Rate of auxin production	0	$[\ \ ]s^{-1}$
d_{auxin}	Rate of auxin degradation	0.0000725	s^{-1}

Supplementary Table 4: Default parameter values for the auxin-AUX1 single cell model.

**Parameter**	**Meaning**	**Value**	**Units**
D_{wall}	Apoplastic auxin diffusion rate	40	$\mu m^2 s^{-1}$
D_{cell}	Cytoplasmic auxin diffusion rate	600	$\mu m^2 s^{-1}$
p_{Aux}	*Rate of auxin production*	*0*	$[\ \ ]s^{-1}$
d_{Aux}	*Rate of auxin degradation*	*0.0000725*	s^{-1}
i_{pas}	Rate of passive auxin influx	2	$[\ \ ]s^{-1}$
i_{aux1}	*Maximum rate of active AUX1 dependent auxin influx*	*0.2*	$\mu m^2 s^{-1}$
$K_{sataux1}$	Intracellular auxin level at which AUX1 influx rate is half maximal	60	$[\ \ ]$
e_{PIN}	*Maximum rate of active PIN1 dependent auxin efflux*	*0.04*	$\mu m^2 s^{-1}$
K_{satpin}	Extracellular auxin leval at which PIN1 efflux rate is half maximal	60	$[\ \ ]$

Supplementary Table 5: Default parameters of the simple cell strand model.

Supplementary Methods

Modelling

Gap frequency model

To investigate whether the observed gap size frequency distributions in *brx* and *pax* mutants are the likely result of a random process or not, we made a simple model of a protophloem cell file in which cells can be either a gap (non-differentiated) or non-gap (differentiated) cell, by varying the chances for individual cells of being a gap cell. Protophloem cell file length was drawn from a range of 6.8 to 10.8 cells, resulting in an average of 8.8 cells, based on our experimental observations (average differentiation zone size of 8.8 for *pax* and 6.7 for *brx* mutants). We compared three model variants:

1) Each cell has the same, independent chance P of being a gap cell. The chance of being a gap cell was set to the fraction of gap cells observed experimentally in *brx* mutants (P=0.18).

2) There is an independent chance P1 for cells to be a gap cell. Additionally there is a chance P2 for a cell to be a gap cell given that the previous cell is a gap cell. P1 and P2 are set such that the resulting fraction of gap cells equals the fraction observed experimentally. Various combinations fulfilling this constraint were investigated (P1=0.1 and P2=0.6; P1=0.07 and P2=0.8; P1=0.05 and P2=0.95).

3) This is a variation on 2. Again there are chances P1 and P2. However, P2 now is the chance for a cell to be a gap cell given that the previous cell is a gap cell and the one before is not a gap cell. Again, P1 and P2 are set such that the resulting fraction of gap cells matches the fraction observed experimentally (P1=0.12 and P2=0.75).

To investigate the gap size frequency distribution each of these scenarios would result in, we randomly generated a total of 100,000 protophloem strands per scenario, quantifying them in the same way as our *in planta* protophloem strands.

Single cell BRX, PAX, PIN model

In this single cell model we assume that overall BRX (BRX_{tot}), PAX (PAX_{tot}) and PIN1 ($PIN1_{tot}$) levels stay constant, and model the dynamics of auxin dependent membrane occupancy of BRX (BRX_{mem}), the auxin dependent phosphorylation of PAX (PAX_p), the BRX- and PAX-dependent phosphorylation of PIN1 ($PIN1_p$), and how average PIN1 efflux rate depends on the fraction of phosphorylated PIN1 using the following set of equations:

$$\frac{dBRX_{mem}}{dt} = brx_{exo}\frac{K_{brx}^2}{K_{brx}^2 + auxin_i^2}(BRX_{tot} - BRX_{mem}) - brx_{endo}BRX_{mem} \qquad \text{Eq. 1}$$

$$\frac{dPAX^P}{dt} = pax_{act}\frac{auxin_i^2}{auxin_i^2 + K_{pax}^2}(PAX_{total} - PAX^p) - pax_{deact}PAX^P \qquad \text{Eq. 2}$$

$$\frac{dPIN1^P}{dt} = pin1_{act}f(PAX, PAX^P)g(BRX_{mem})(PIN1_{total} - PIN1^p) - pin1_{deact}PIN1^P \qquad \text{Eq. 3}$$

$$f(PAX, PAX^P) = \frac{(PAX_{total} - PAX^p)^2 + enh_{paxp}PAX^{p^2}}{(PAX_{total} - PAX^p)^2 + enh_{paxp}PAX^{p^2} + K_{PIN1,PAX^P}^2} \qquad \text{Eq. 3b}$$

$$g(BRX_{mem}) = \frac{K_{Pin1,brx}^2}{K_{Pin1,brx}^2 + BRX_{mem}^2} \qquad \text{Eq. 3c}$$

$$pinexport = ppump\frac{(PIN1_{total} - PIN1^p) + enh_{pinp}PIN^P}{enh_{pinp}} \qquad \text{Eq. 4}$$

Note that we kept intracellular auxin as a control parameter external to the model to investigate the effect of auxin levels on PIN1-mediated export. In the multi-cellular, 1D strand models, intra- and extracellular auxin dynamics were modelled as independent variables (see below).

Note that in the equation for BRX_{mem}, the negative effect of auxin on BRX_{mem} levels is incorporated through a repression of BRX exocytosis. The alternative, auxin-induced BRX endocytosis would lead to similar outcomes. In the equation for PAX_p, we incorporated that PAX phosphorylation rate is enhanced in the presence of auxin. For the equation of PIN_p, we incorporated the PAX dependence of PIN phosphorylation ($f(PAX,PAX_p)$), and the enhanced PIN (enhpaxp>1) phosphorylation potential of phosphorylated PAX. Additionally, for PIN_p we incorporate the repression of PAX mediated phosphorylation by BRX_{mem} ($g(BRX_{mem})$. Finally, average PIN transport rate depends on the fraction of highly active (enhpinp>1) phosphorylated and less active, non-phosphorylated PINs.

With regard to parameter values, experimental data suggest a 2-3 fold increase in auxin efflux due to PIN phosphorylation[1], thus providing us with a ballpark figure for the value of parameter enh_{pinp}. For other parameters, no quantitative experimental data were available and robustness of results against variation in parameter values was investigated. The qualitative shape of the dependence of PIN1 efflux rate on auxin levels remained the same for 2-fold changes in the parameters controlling the efficiency of PAX versus PAX_p in PIN phosphorylation and of PIN versus PINp in transporting auxin, as well as 33% increases or decreases in saturation constants. See Supplementary Table 3 for an explanation of parameter meaning, default values and units.

Total cellular levels of BRX, PAX and PIN proteins (BRX_{tot}, PAX_{tot}, $PIN1_{tot}$) were normalized to an arbitrary value of 100. Ratios between BRX exo- and endocytosis rates (brx_{exo}, brx_{endo}) as well as PAX (pax_{act}, pax_{deact}) and PIN ($pin1_{act}$,$pin1_{deact}$) phosphorylation and dephosphorylation rates were set to 10 to ensure that under optimal conditions (very low auxin; very high auxin; and very low BRX_{mem} and high PAX_p, respectively) a near to 100% (10/11) fraction of BRX can reside on the membrane, or PAX and PIN can be phosphorylated, respectively. In addition to choosing this ratio, rates are set such that they exceed the rates of *AUX1* and *PIN1* gene expression used later in described cell strand models. Saturation constants for auxin dependent BRX endocytosis K_{brx} and auxin dependent PAX phosphorylation K_{pax}were (because of the opposing effects of BRX_{mem} and PAX_p on PIN_p levels) choosen to lie near the opposite ends of the auxin range occuring in our models. Additionally, saturation constants mediating the effects of BRX_{mem} and PAX_p on PIN phosphorylation rate ($K_{Pin1,brx} K_{PIN1,pax}$) were set such that BRX_{mem} starts having an effect at low levels, whereas for PAX effects to become strong considerably higher concentrations are needed. Combined this ensures that PIN_p levels can gradually vary over a wide range of auxin levels, enabling effective BRX-PAX-PIN-mediated auxin homeostasis. As explained above enh_{pinp} is based on experimental data and enh_{pax} is choosen to have a similar order of magnitude.

Changes in membrane occupancy of BRX, as well as in PAX and PIN phosphorylation levels will occur rapidly relative to changes in transcription, as well as processes of cell growth, division and expansion. Therefore, rather than using the dynamic equations 1-3 we used a quasi-steady-state assumption, enabling us to apply the following algebraic expressions:

$$BRX_m = \frac{BRX_{total}}{1+\frac{brx_{endo}}{brx_{exo}\frac{K_{brx}^2}{K_{brx}^2+auxin_i^2}}} \quad \text{Eq. 5}$$

$$PAX^p = \frac{PAX_{total}}{1+\frac{pax_{inact}}{pax_{act}\frac{auxin_i^2}{auxin_i^2+K_{pax}^2}}} \qquad \text{Eq. 6}$$

$$PIN1_p = \frac{PIN1_{total}}{1+\frac{pin_{deact}}{pin_{act}f(PAX,PAX^p)g(BRX_m)}} \qquad \text{Eq. 7}$$

brx mutants were simulated by setting $BRX_{total} = 0$, while pax mutants were simulated by setting $PAX_{total} = 0$.

Single cell AUX1 model

In this single cell model we solely focused on the auxin-dependence of AUX1 expression and its effect on intracellular auxin levels $(auxin_i)$. Extracellular auxin levels $(auxin_e)$ are set constant and are used as a control parameter. PIN1 phosphorylation and its effect on PIN1 efflux rate are ignored. Based on our observation of a positive relation between auxin and AUX1 protein levels as well as earlier results[2], we modeled a positive effect of auxin on AUX1 protein production:

$$\frac{\mathrm{dAUX1}}{\mathrm{dt}} = \mathrm{p}_{\mathrm{AUX1}} \frac{{auxin_i}^2}{{auxin_i}^2+\mathrm{K}_{\mathrm{AUX1}}^2} - d_{\mathrm{AUX1}}\mathrm{AUX1} \qquad \text{Eq. 8}$$

Note that incorporating a negative effect of auxin on AUX1 degradation instead would yield similar results.

We initially make the simplifying assumption that PIN1 and AUX1 auxin transport increases linearly with levels of available auxin. Together this results in the following auxin dynamics:

$$\frac{dauxin_i}{\mathrm{dt}} = \mathrm{i}_{\mathrm{aux1}}\mathrm{AUX1}auxin_e - e_{\mathrm{pin}}\mathrm{PIN1}auxin_i + p_{\mathrm{auxin}} - d_{\mathrm{auxin}}auxin_i \qquad \text{Eq. 9}$$

See Supplementary Table 4 for an explanation of parameter meaning and default values.

Rate of AUX1 mediated influx i_{aux1} and auxin degradation d_{auxin} are similar as in previous models[2,3], while for simplicity (given the dominance of shoot and reflux loop-derived auxin) local auxin production p_{aux1} is ignored. PIN1-mediated efflux rate e_{pin1} was reduced 5-fold relative to previous models, to prevent all auxin from accumulating in the lowermost cell. AUX1 production p_{aux1} and decay rates d_{aux1} were taken to result in a maximum expression level of 100, equal to the total amount of $PIN1$ present in a cell. Additionally, values were taken such that AUX1 transcriptional and translational dynamics are slower than BRX endo/exocytosis and PAX/PIN phosphorylation/dephosphorylation. The saturation constant for AUX1 expression K_{aux1} was set to approximately halfway the maximally occurring auxin values.

Applying steady state assumptions this results in the following equations for the auxin and AUX1 null-clines:

$$AUX1 = \frac{e_{pin}\frac{p_{PIN1}}{d_{PIN1}}+d_{auxin}}{i_{AUX1}auxin_e} auxin_i - \frac{p_{auxin}}{i_{AUX1}auxin_e} \qquad \text{Eq. 10}$$

$$AUX1_{ss} = \frac{p_{AUX1}}{d_{AUX1}}\frac{{auxin_i}^2}{{auxin_i}^2+K_{AUX1}^2} \qquad \text{Eq. 11}$$

This enabled us to draw the phase plane diagram. The auxin nullcline is a straight line. Assuming that auxin transport rates exceed auxin production and degradation rates, the slope approximates $\frac{e_{pin}}{i_{AUX1}}\frac{PIN1_{ss}}{auxin_e}$, and the negative intercept $\frac{p_{auxin}}{i_{AUX1}auxine_e}$ will be very small (we used $p_{auxin} = 0$ resulting in an intercept at zero). The AUX1 null-cline corresponds to a second order Hill function that

approaches $\frac{p_{AUX1}}{d_{AUX1}}$ for large values of auxin, and its point of half maximum occurs for auxin equal to K_{AUX1}. For all parameter values, the two null-clines will intersect at a point close to (auxin=0,AUX1=0) (or at this point for $p_{auxin} = 0$), which corresponds to a stable state of the system. If the slope of the auxin null-cline is high, only this single intersection point will occur, resulting in a single low auxin-low AUX1 equilibrium. For parameter values resulting in a more shallow slope of the auxin null-cline two additional intersection points arise, the higher one of which is an alternative high auxin-high AUX1 stable state, and the lower one an unstable state separating the two stable equilibria. Finally, if parameter values result in very shallow slopes, although the lower stable equilibrium remains present, the unstable equilibrium will lie infinitesimally nearby, enabling the system to only converge to the higher stable equilibrium.

Note that if we instead assume that PIN1 and AUX1 auxin transport are not proportionate to auxin levels, but saturate with increasing auxin levels (as we did for the spatial, 1D cell strand models described below) the auxin equation changes into:

$$\frac{dauxin}{dt} = i_{AUX1}AUX1\frac{auxin_e}{auxin_e+K_e} - e_{pin1}PIN1\frac{auxin}{auxin+K_i} + p_{auxin} - d_{auxin}auxin \qquad \text{Eq. 12}$$

This changes the equation for the auxin null-cline into:

$$AUX1 = \frac{e_{pin}PIN1\frac{auxin}{auxin+K_i}}{i_{AUX1}\frac{auxin_e}{auxin_e+K_e}} + \frac{d_{auxin}}{i_{AUX1}\frac{auxin_e}{auxin_e+K_e}}auxin - \frac{p_{auxin}}{i_{AUX1}\frac{auxin_e}{auxin_e+K_e}} \qquad \text{Eq. 13}$$

Since external auxin is a control parameter, the saturation terms with $auxin_e$ in them merely represent constants, with higher external auxin values resulting in division by a larger number. Thus, this leaves only the new saturating dependence on internal auxin as being relevant for null-cline shape, and we see that the auxin null-cline now depends on internal auxin levels with both a saturating and a linear dependence term, whereas earlier we only had a linear dependence. Despite this change in null-cline shape we still get the following sequence of situations for increasing external auxin: For low external auxin (division by small number) this auxin null-cline intersects the AUX1 null-cline only in a single low auxin low AUX1 point, overshooting it for higher internal auxin values. If instead external auxin is higher (division by larger number), the null-clines intersect three times resulting in a low and high stable equilibrium separated by an instable intermediate equilibrium. Finally, if external auxin levels are even higher (division by very large number) the lower and middle intersection points lie very close together, and only the high auxin high AUX1 equilibrium is accessible. Thus, qualitative model behavior remains the same. Null-cline and bifurcation analysis was performed using Grind in R, developed by R. De Boer at Utrecht University (*http://theory.bio.uu.nl/rdb/grind.html*).

Simple cell strand model

This model describes a simple strand of 15 cells, ignoring cell growth, division, expansion and differentiation processes and the resulting zonation. The model incorporates for each individual cell the BRX_m, PAX_p, and PIN_p dynamics as well as the AUX1 dynamics described in the previous two single cell models. Additionally, the model dynamically describes intracellular and extracellular auxin dynamics, in a manner similar to previous models[2-5].

For a grid point i,j inside the wall bordered by only wall grid points we write:

$$\frac{\delta \mathrm{Aux}_{i,j}}{\delta t} = \frac{D_{\mathrm{wall}}}{\Delta x}\left(\mathrm{Aux}_{i+1,j}+\mathrm{Aux}_{i-1,j}+\mathrm{Aux}_{i,j+1}+\mathrm{Aux}_{i,j-1} - 4\mathrm{Aux}_{i,j}\right) \qquad \text{Eq. 14}$$

Here D_{wall} is the diffusion rate for auxin in the apoplast and Δxis the spatial resolution of the simulation. For a grid point i,j inside the cytoplasm bordered by only other cytoplasmic grid points we write:

$$\frac{\delta \text{Aux}_{\text{i,j}}}{\delta \text{t}} = \text{p}_{\text{Aux}} - d_{\text{Aux}}\text{Aux}_{\text{i,j}} + \frac{D_{\text{cell}}}{\Delta \text{x}}\left(\text{Aux}_{\text{i+1,j}} + \text{Aux}_{i-1,\text{j}} + \text{Aux}_{\text{i,j+1}} + \text{Aux}_{\text{i,j}-1} - 4\text{Aux}_{\text{i,j}}\right) \qquad \text{Eq. 15}$$

Here p_{Aux} is the rate at which auxin is produced per cell, d_{Aux} is the rate at which auxin is degraded per cell, and D_{cell} is the diffusion rate for auxin inside cells.

For a grid point i,j inside the wall, bordered by three other wall grid points and one cytoplasmic grid point (i,j-1) and hence membrane grid point we write:

$$\frac{\delta \text{Aux}_{\text{i,j}}}{\delta \text{t}} = -i_{pas} - i_{\text{aux1}}AUX1_{i,j}f\left(\text{Aux}_{\text{i,j}}\right) + \text{e}_{\text{PIN}}PIN_{i,j}g\left(\text{Aux}_{\text{i,j}-1}\right) + \frac{D_{\text{wall}}}{\Delta \text{x}}\left(\text{Aux}_{\text{i+1,j}} + \text{Aux}_{i-1,\text{j}} + \text{Aux}_{\text{i,j+1}} - 3\text{Aux}_{\text{i,j}}\right) \qquad \text{Eq. 16}$$

with $f\left(Aux_{i,j}\right) = \frac{Aux_{i,j}}{Aux_{i,j}+K_{sataux1}}$ and $g\left(Aux_{i,j}\right) = \frac{Aux_{i,j}}{Aux_{i,j}+K_{satpin}}$

Here, i_{pas} is the rate of passive auxin influx from walls to cytoplasm, i_{aux1} is the maximum rate of active auxin influx through AUX1 from walls to cytoplasm, $K_{sataux1}$ is the internal auxin level at which AUX1 operates at its half maximum rate, e_{PIN} is the maximum rate of active pumping of auxin through PINs from cytoplasm to walls, and K_{satpin} is the external auxin level at which PIN operates at its half maximum rate. Finally, for a point i,j inside the cytoplasm, neighboring three other cytoplasmic grid points and one cell wall grid point (i,j-1) we write:

$$\frac{\delta \text{Aux}_{\text{i,j}}}{\delta \text{t}} = i_{pas} + \text{i}_{\text{aux1}}AUX1_{i,j}f\left(\text{Aux}_{\text{i,j}-1}\right) - \text{e}_{\text{PIN}}PIN_{i,j}g\left(\text{Aux}_{\text{i,j}}\right) + \frac{D_{\text{cell}}}{\Delta \text{x}}\left(\text{Aux}_{\text{i+1,j}} + \text{Aux}_{i-1,\text{j}} + \text{Aux}_{\text{i,j+1}} - 3\text{Aux}_{\text{i,j}}\right) \qquad \text{Eq. 17.}$$

Note that auxin dynamics are solved on a subcellular grid level, incorporating both transmembrane transport processes as well as intracellular and intra-wall diffusional auxin transport, similar to earlier models[2,3]. Note as well that the amount of auxin transported by a single PIN or AUX1 protein saturates with increasing levels of auxin, something that is usually ignored. Finally note that e_{PIN} is not a parameter, but instead a constant rate parameter multiplied by a term that is dependent on the fraction of phosphorylated PIN (Eq. 4).

Based on experimental data from protophloem cells, we assume a highly rootward polarized PIN1 pattern, with limited auxin exporter levels at lateral and shootward membrane faces (10% of downward oriented PIN1). Also based on our experimental data, we assume an apolar AUX1 pattern, with AUX1 levels highest at the apical and basal membranes, and lower (50%) at the lateral membranes. Finally, we incorporate influx of auxin at the top of the cell file (applying $p_{aux} = 10[\quad]s^{-1}$ in the top-most horizontal cell wall), a small auxin influx at the sides of the cell file (applying $p_{aux} = \frac{10}{tissuelength}[\quad]s^{-1}$ for each lateral cell wall grid point), as well as an auxin efflux at the bottom of the cell file (applying $d_{aux} = 0.25s^{-1}$ in the bottom-most horizontal cell wall).

Parameter settings for auxin dynamics can be found in Supplementary Table 5. Auxin dynamics were solved using an alternating direction implicit (ADI) integration scheme[6], using a timestep of 0.2ms and a spacestep of 2 micrometer.

Parameter values for auxin diffusion D_{wall} and D_{cell}, and passive influx i_{pas} are similar to those of earlier studies[2,3]. Saturation constants for AUX1 and PIN1 transport rates $K_{sataux1}$ and K_{satpin} were set to approximately halfway the maximally observed auxin concentrations. e_{PIN} was set to a 5 times lower value than in our previous models based on our recent observation of donut-shaped PIN1 patterns in protophloem cells, which effectively reduces PIN1-mediated auxin export[7]. Other parameters (in italics) were already discussed in Table 2.

For the simulations with BRX_{mem}, PAX_p, and PIN_p dynamics disabled, a constant level of PIN_p=60 is assumed, and auxin influx from the top is varied. For the simulations with full BRX_{mem}, PAX_p, and PIN_p model dynamics a constant auxin influx from the top is applied.

Developing protophloem strand model

As a final step we extended the simple, static single strand model to a realistic, zonated and growing protophloem sieve element (PPSE) strand model.

Zonation

We built up our PPSE strand model out of 2 distinct zones: the meristematic and the differentiation zone. Additionally, within the meristem we distinguish the three most rootward cells as being slower dividing stem cells, consistent with experimental observations. Through division, individual cells will sequentially move towards and into the differentiation zone, being pushed shootward by cells newly arising from stem cell divisions as well as the expansion of existing clones lying rootward. For simplicity, we superimposed the location of the meristematic-differentiation zone boundary, with the meristematic to differentiation zone boundary at a distance of on average 15 meristematic cells from the start of the PPSE strand (since cells change size due to growth and division processes, this results in an average number of cells fitting in a constant-sized domain) and setting overall cell file length such that a differentiation zone containing an average of 6-8 cells with a maximum size of 30 micrometer arises (consistent with experimental evidence). Note that we did not explicitly incorporate the elongation zone in our PPSE strand model. In this zone, cells have become fully differentiated, and in the case of protophloem cells this means strongly reduced organelles and fully enucleated, with minimal resistance to phloem sap transport. Therefore, while auxin transport in the meristem and differentiation zone is governed by AUX1 importer and PIN exporter levels and activities, auxin transport in the differentiation zone mainly occurs through bulk flow. Therefore, this part of the auxin dynamics was represented as auxin inflow into the differentiation zone.

Growth, division and expansion

Division and elongation rates: We assume that normal meristematic cells have a division time of 12 hours, consistent when correcting classical average cell cycle measurements on the entire Arabidopsis root meristem[8] with the more recent observation that a substantial rootward part of the meristem does not take part in active divisions[9,10]. The applied expansion rate is the same as in earlier models[11]. We assume that the three most rootward oriented stem cell that divide at rates that are slower than the normal meristematic cells yet increase shootward, consistent with experimental observations[11]. We assume division in the lower-most stem cell to be so slow that we ignore them, we assume a division time of 25.2 hours for the second stem cell, and a division time of 16.8 hours for the third stem cell. These numbers were chosen such that they are neither multiples of one another nor of the normal meristematic cells, ensuring the generation of out-of-phase clones of sibling cells, also consistent with experimental observations[12].

Division and elongation on a grid: Cell growth and expansion is modeled as in Mahonen et al., 2014[3]. Briefly, individual cells consist of a number of rows and columns of grid points. If a cell undergoes either cytoplasmic growth or vacuolar expansion, a row of gridpoints is added to the shootward part of the cell and all rootward lying cells are shifted one row upward on the simulation grid. Auxin concentrations are diluted to compensate for the resulting instantaneous cellular volume increase, protein concentrations are only diluted in case of cytoplasmic, but not vacuolar volume increase. Cell division occurs if cells have reached twice their original size (increase from 8 to 16 micrometer). Upon cell division, cells inherit the PIN and AUX1 patterns and levels of their maternal cell. During expansion, cells can reach a maximum size of 30 micrometer, consistent with the sizes reached within the differentiation zone. Cell behavior (cytoplasmic growth and division, elongation) is dictated by the zone in which a cell resides (see previous section).

Finite grid size: Cells exceeding a threshold position from the start of the simulated PPSE strand with their apical membrane are removed from the simulation to prevent a continuous increase in the size of the simulated domain. The threshold distance is taken such that at least 5 cells in the elongation zone are contained within the simulation.

Reflux loop emulation

To correctly simulate protophloem auxin patterning, we take into account that in addition to auxin received from the auxin flux via shootward differentiated phloem, and auxin loss to more rootward tissues, auxin is also received laterally through the presence of the root tip auxin reflux loop[5], and that this results in a predominant recycling of auxin at the top of the meristem, with influx gradually decreasing when moving closer to the root tip (see illustration for p_{aux}values (black) and d_{aux}values (red) used to emulate this reflux influx and efflux, respectively). For lateral auxin flow into the meristem, we furthermore apply:

$$influx_j = \left(a_{pinp}\, PINp/PINtot + \left(1 - a_{pinp}\right)\right)\left(b_{basal} + b_{increase}\, j/MZsize\right)$$

Where b_{basal} is the baseline influx rate occurring at the most rootward positions, and $b_{increase}$ (set at twice the value of b_{basal}) the maximum increase in influx rate occurring at the end of the meristem (see Supplementary Figure 8), and a_{pinp} is the phosphorylated PIN-dependent and $\left(1 - a_{pinp}\right)$ the phosphorylated PIN-independent fraction. Thus, we take into account that *brx* and *pax* mutations that affect PIN phosphorylation level, through affecting root tip auxin delivery, will also affect auxin reflux into the protophloem strand itself. Under default conditions $a_{pinp} = 0.65$, only in case of non-auxin-dependent AUX1 expression we use $a_{pinp} = 1$ to compensate for the higher overall AUX1 levels in absence of auxin-dependent AUX1. Finally, we assume that efflux at the bottom of the protophloem strand results in a maximum auxin level (put to 150).

For the simulation results where auxin-dependent AUX1 expression was disabled, a constant AUX1 expression level of 100 was assumed. Otherwise the normal auxin-dependent AUX1 expression was applied.

Adding Differentiation

To investigate the impact of the timing of auxin-dependent protophloem cell differentiation on gap patterning, we implemented two alternative differentiation scenarios in our model. In both scenarios the increase in differentiation level as a function of auxin is modelled as:

$$\frac{dDiff}{dt} = p_{diff}\frac{{auxin_i}^2}{{auxin_i}^2 + K_{Diff}^2} \qquad \text{Eq. 18}$$

With p_{diff} the maximum rate of differentiation level increase, and $K_{Diff}$the auxin level at which this rate is half maximal. Because of the considerably higher auxin levels in the division zone as compared to more shootward zones as well as the longer time cells spend in this zone, we use $K_{Diff} = 75[\ \ ]s - 1$and$p_{diff} = 0.002[\ \ ]s^{-1}$ for the early differentiation scenario, and $K_{Diff} = 35[\ \ ]s^{-1}$ and $p_{diff} = 0.004[\ \ ]s^{-1}$ for the late differentiation scenario, to ensure that we are investigating differences in the timing of differentiation onset and not final differences in the differentiation level reached. Note that the differentiation dynamics equation contains no decay term. This serves to ensure that once auxin levels drop no de-differentiation will occur. If a differentiation level of 100 is reached, no further increase in differentiation is modeled.

Early starting-early ending differentiation ("early scenario")

In this scenario, the equation for differentiation dynamics shown above is applied from 6 cell heights rootward of the bottom of the simulated PPSE strand until the end of the meristematic zone.

Late starting-late ending differentiation ("late scenario")

In this scenario, the equation for differentiation dynamics shown above is applied from the start of the differentiation zone onwards.

Auto-activation of differentiation

We assume that once a certain threshold level of the simulated differentiation state is reached, cells will proceed to successfully differentiate into a protophloem cell, while cells failing to reach this level will become non-differentiated gap cells. Such dynamics will automatically arise if the differentiation process, in addition to being auxin-dependent, also has a positive feedback on itself. To explicitly simulate this, in a subset of simulations we replaced the equation for the differentiation factor with:

$$\frac{dDiff}{dt} = p_{diff} max\left(\frac{auxin_i^2}{auxin_i^2 + K_{Diff}^2}, \frac{Diff^4}{Diff^4 + K_{m,diff}^4}\right), \qquad \text{Eq. 18b}$$

where *max* indicates a maximum function, effectively implementing an OR-type logic for *the auxin* and *Diff* influence on *Diff* expression, and $K_{m,diff} = 65$ the level of *Diff* at which half-maximal auto-activation occurs. Importantly, while auxin-dependent differentiation occurs within a restricted spatio-temporal window (either early or late scenario), no such restrictions apply for auto-activated differentiation.

Adding YUCCA

To investigate the potential effect of local rather than shoot-derived auxin on protophloem differentiation, we incorporated the *YUC5* expression. Based on the observation that *YUC5* is only expressed in the differentiation zone, but not in the meristematic zone or stem cell niche, we assumed that *YUC5* expression depends on differentiation status. We therefore model *YUC5* expression as:

$$\frac{dYUC5}{dt} = p_{yucr} \frac{Diff^2}{Diff^2 + K_{yuc5}^2} - d_{yurc} YUC5 \qquad \text{Eq. 19}$$

With $p_{yuc5} = 0.01s^{-1}$ the maximum rate of *YUC5* expression, $K_{yuc5} = 65[\ \ ]$ the differentiation level at which *YUC5* expression is half maximal, and $d_{yuc5} = 0.0001s^{-1}$ the degradation rate of YUC5.

We assume that YUC5 expression has the following effect on local auxin biosynthesis:

$$p_{aux} = 0.001YUC5$$

To investigate the dependence on the *source* of auxin rather than the *absolute level* of auxin, we computed the extra amount of auxin present in a wildtype simulation as compared to when no YUCCA-mediated local auxin production was incorporated. We subsequently reduced the amount of shoot-derived auxin influx to arrive at a similar amount of overall auxin as before.

Robustness of results for the simple single strand model and the developing protophloem strand model were tested both against the parameter variations also used to test robustness of the single cell protophloem model, as well as against variations in the precise AUX1 cellular pattern. For the latter, in addition to the default pattern in which basal and apical membranes have more AUX1 than the lateral membranes, also a fully apolar pattern with equal AUX1 levels on all membrane faces, and a more polar pattern with highest AUX1 levels on the apical surface were tested. In all cases only small quantitative differences were observed, while qualitative outcomes remained constant.

Source codes for the different models described here are available on:

http://bioinformatics.bio.uu.nl/khwjtuss/ProtophloemModel

Supplemental References:

1 Marhava, P. *et al.* A molecular rheostat adjusts auxin flux to promote root protophloem differentiation. *Nature* **558**, 297-300, doi:10.1038/s41586-018-0186-z (2018).

2 van den Berg, T., Korver, R. A., Testerink, C. & ten Tusscher, K. H. W. J. Modeling halotropism: a key role for root tip architecture and reflux loop remodeling in redistributing auxin. *Development* **143**, 3350-3362, doi:10.1242/dev.135111 (2016).

3 Mahonen, A. P. *et al.* PLETHORA gradient formation mechanism separates auxin responses. *Nature* **515**, 125-129, doi:10.1038/nature13663 (2014).

4 Mitchison, G. J. Model for Vein Formation in Higher-Plants. *Proc R Soc Ser B-Bio* **207**, 79-109, doi:DOI 10.1098/rspb.1980.0015 (1980).

5 Grieneisen, V. A., Xu, J., Maree, A. F., Hogeweg, P. & Scheres, B. Auxin transport is sufficient to generate a maximum and gradient guiding root growth. *Nature* **449**, 1008-1013, doi:10.1038/nature06215 (2007).

6 Peaceman, D. W. & Rachford, H. H. The Numerical Solution of Parabolic and Elliptic Differential Equations. *J Soc Ind Appl Math* **3**, 28-41, doi:Doi 10.1137/0103003 (1955).

7 Marhava, P. *et al.* Plasma Membrane Domain Patterning and Self-Reinforcing Polarity in Arabidopsis. *Dev Cell* **52**, 223-235, doi:10.1016/j.devcel.2019.11.015 (2020).

8 Beemster, G. T. S. & Baskin, T. I. Analysis of cell division and elongation underlying the developmental acceleration of root growth in Arabidopsis thaliana. *Plant Physiol* **116**, 1515-1526, doi:DOI 10.1104/pp.116.4.1515 (1998).

9 Ivanov, V. B. & Dubrovsky, J. G. Longitudinal zonation pattern in plant roots: conflicts and solutions. *Trends in Plant Science* **18**, 237-243, doi:10.1016/j.tplants.2012.10.002 (2013).

10 Pacheco-Escobedo, M. A. *et al.* Longitudinal zonation pattern in Arabidopsis root tip defined by a multiple structural change algorithm. *Ann Bot-London* **118**, 763-776, doi:10.1093/aob/mcw101 (2016).

11 Campilho, A. *et al.* Time-lapse analysis of stem-cell divisions in the Arabidopsis thaliana root meristem. *Plant Journal* **48**, 619-627, doi:10.1111/j.1365-313X.2006.02892.x (2006).

12 von Wangenheim, D. *et al.* Live tracking of moving samples in confocal microscopy for vertically grown roots. *Elife* **6**, e26792 doi:10.7554/eLife.26792 (2017).
